# Spatio‐temporal changes in chimpanzee density and abundance in the Greater Mahale Ecosystem, Tanzania

**DOI:** 10.1002/eap.2715

**Published:** 2022-09-30

**Authors:** Joana S. Carvalho, Fiona A. Stewart, Tiago A. Marques, Noemie Bonnin, Lilian Pintea, Adrienne Chitayat, Rebecca Ingram, Richard J. Moore, Alex K. Piel

**Affiliations:** ^1^ School of Biological and Environmental Sciences Liverpool John Moores University Liverpool UK; ^2^ School of Built and Natural Sciences University of Derby Derby UK; ^3^ Greater Mahale Ecosystem Research and Conservation Project Dar es Salaam Tanzania; ^4^ Department of Anthropology University College London London UK; ^5^ School of Mathematics and Statistics University of St. Andrews St. Andrews UK; ^6^ Department of Animal Biology Faculdade de Ciencias da Universidade de Lisboa Lisbon Portugal; ^7^ Department of Conservation Science The Jane Goodall Institute Washington District of Columbia USA; ^8^ Institute of Biodiversity and Ecosystem Dynamics University of Amsterdam Amsterdam Netherlands

**Keywords:** conservation, density surface modeling, detection function estimation, eastern chimpanzee, generalized additive models, great apes, line‐transect distance sampling, spatially explicit models

## Abstract

Species conservation and management require reliable information about animal distribution and population size. Better management actions within a species' range can be achieved by identifying the location and timing of population changes. In the Greater Mahale Ecosystem (GME), western Tanzania, deforestation due to the expansion of human settlements and agriculture, annual burning, and logging are known threats to wildlife. For one of the most charismatic species, the endangered eastern chimpanzee (*Pan troglodytes schweinfurthii*), approximately 75% of the individuals are distributed outside national park boundaries, requiring monitoring and protection efforts over a vast landscape of various protection statuses. These efforts are especially challenging when we lack data on trends in density and population size. To predict spatio‐temporal chimpanzee density and abundance across the GME, we used density surface modeling, fitting a generalized additive model to a 10‐year time‐series data set of nest counts based on line‐transect surveys. The chimpanzee population declined at an annual rate of 2.41%, including declines of 1.72% in riparian forests (from this point forward, forests), 2.05% in miombo woodlands (from this point forward, woodlands) and 3.45% in nonforests. These population declines were accompanied by ecosystem‐wide declines in vegetation types of 1.36% and 0.32% per year for forests and woodlands, respectively; we estimated an annual increase of 1.35% for nonforests. Our model predicted the highest chimpanzee density in forests (0.86 chimpanzees/km^2^, 95% confidence intervals (CIs) 0.60–1.23; as of 2020), followed by woodlands (0.19, 95% CI 0.12–0.30) and nonforests (0.18, 95% CI 0.10–1.33). Although forests represent only 6% of the landscape, they support nearly one‐quarter of the chimpanzee population (769 chimpanzees, 95% CI 536–1103). Woodlands dominate the landscape (71%) and therefore support more than a half of the chimpanzee population (2294; 95% CI 1420–3707). The remaining quarter of the landscape is represented by nonforests and supports another quarter of the chimpanzee population (750; 95% CI 408–1381). Given the pressures on the remaining suitable habitat in Tanzania, and the need of chimpanzees to access both forest and woodland vegetation to survive, we urge future management actions to increase resources and expand the efforts to protect critical forest and woodland habitat and promote strategies and policies that more effectively prevent irreversible losses. We suggest that regular monitoring programs implement a systematic random design to effectively inform and allocate conservation actions and facilitate interannual comparisons for trend monitoring, measuring conservation success, and guiding adaptive management.

## INTRODUCTION

The interaction among threats such as drivers of deforestation, overharvesting of wildlife, and climate change is leading to species and wildlife population declines and extinctions, particularly in the tropics (Barlow et al., 2018; Dirzo et al., 2014). A decline of 4.1% in global forest cover was reported between 1960 and 2019, with the highest rates of land‐use change and degradation found in the tropics, where deforestation exceeded 0.8 million/km^2^ (Winkler et al., 2021). This is of urgent concern given that the remaining tropical forests are increasingly valuable for biodiversity, providing important ecosystem services at global and local scales (Edwards et al., 2019).

Along with other primates, great apes (bonobos, chimpanzees, gorillas, and orangutans) are particularly vulnerable to anthropogenic threats due to their low reproductive rate, late age of first birth, long interbirth intervals, and low population densities (Purvis et al., 2000). A considerable reduction in the area of suitable environmental conditions over the past 20 years as well as large population losses caused by anthropogenic activities and/or disease epidemics are well documented in great apes (Junker et al., 2012; Kuehl et al., 2017; Plumptre et al., 2016; Strindberg et al., 2018). This is despite the fact that they show a certain behavioral flexibility enabling them to adapt and survive in human‐modified habitats (Heinicke et al., 2019).

Tanzania represents the eastern‐most boundary of chimpanzee distribution, where the eastern chimpanzee (*P. t. schweinfurthii*) is classified as Endangered on the IUCN Red List (Humle et al., 2016). Most chimpanzees occur in the GME, western Tanzania, which hosts ~90% of the country's estimated 2000–3000 chimpanzees (Moyer et al., 2006), most of which live outside of national park boundaries (Plumptre et al., 2010). Because of the savanna–woodland mosaic habitat that comprises the GME, chimpanzees are distributed across various vegetation types, from open grasslands with scattered trees to woodlands and dense riverine forest strips. They occur at low densities and exhibit comparatively large home ranges (Giuliano et al. unpublished data; Kano, 1971; Ogawa et al., 2006; Piel & Stewart, 2014; Piel et al., 2015). Specific threats to Tanzania's chimpanzees include habitat loss due to the expansion of human settlements, smallholder agriculture and roads, uncontrolled fire, charcoal production, logging, livestock expansion, commercial timber use and anthropogenic disease (Ogawa et al., 2006; Piel et al., 2015; Plumptre et al., 2010; TAWIRI, 2018). Despite being the least available vegetation type, forests offer high quality feeding and other suitable conditions for chimpanzees across the GME (Bonnin et al., 2020) and have been reported to have dramatically higher densities than woodlands (e.g., 0.29 vs. 0.04 chimpanzees/km^2^, Piel et al., 2015). However, forests are composed of rich soils and often provide year‐round water‐features that are also highly desirable for cultivation and are therefore threatened due to the expansion of agriculture (Ogawa et al., 2006; Pintea et al., 2021; TAWIRI, 2018). Moreover, expansion of human settlements occurs particularly close to forests (Chitayat et al., 2021; Ogawa et al., 2013; Pintea et al., 2021; Plumptre et al., 2010), posing an additional threat to chimpanzee distribution and survival, and to the effectiveness of conservation efforts in this important ecosystem.

Robust estimates of population density and abundance are crucial for determining the conservation status of species, and are essential for implementing effective conservation and management policies (Marques et al., 2013). Distance sampling (Buckland et al., 2015) is a widely used technique for estimating density and abundance, particularly from line‐transect and point‐transect data collected by human observers, cameras, or acoustic devices (Buckland et al., 2010; Howe et al., 2017; Marques et al., 2013). Density estimates are obtained using a hybrid of design‐ and model‐based methods. Design‐based methods extrapolate densities from each survey stratum to the stratum area based on the properties of a random sampling design (Buckland et al., 2015). Alternatively, if the goal is to map species distribution or compare densities at different spatial scales, densities from distance sampling surveys can also be inferred from a model‐based approach (Buckland et al., 2000; Miller et al., 2013).

Density surface modeling (DSM) is a model‐based method that estimates density and uncertainty from distance sampling data, and extrapolates density to broader areas by accounting for the influence of key spatial variables on density (Hedley & Buckland, 2004; Miller et al., 2013). DSM is traditionally a two‐stage approach that (1) fits a detection function to the distance data to obtain detection probabilities for groups or individuals, and (2) employs a generalized additive model (GAM; Wood, 2017) that integrates count data per transect segment corrected for detectability as a smooth function of important environmental variables. DSM has provided useful information to decision makers regarding spatio‐temporal patterns of abundance and density of both marine (Roberts et al., 2016) and terrestrial species (Camp et al., 2020; Dias et al., 2019).

Several studies have provided chimpanzee density and abundance estimates for specific regions of the GME to date using a design‐based approach (Moore & Vigilant, 2014; Moyer et al., 2006; Ogawa et al., 2006, 2013; Piel et al., 2015; Suzuki, 1969; Yoshikawa et al., 2008). However, no assessment of chimpanzee population size across time and space has yet been made from a model‐based approach, containing a large temporal and spatial coverage of chimpanzee surveys and relevant environmental predictors. Fitting innovative spatially explicit models, we estimated densities and abundances based on a 10‐year time series of nest surveys of chimpanzees across the GME. Specifically, we used DSMs to (1) make inferences about chimpanzee population size between 2010 and 2020; (2) relate these estimates to relevant environmental factors; and (3) predict spatio‐temporal changes in density and abundance in the study area. Specifically, we addressed the following questions: (1) How many chimpanzees occur across the GME? (2) What is the annual trend in population size? (3) What is the annual trend in cover of different vegetation types? (4) How do chimpanzee densities differ among vegetation types over time? (5) How do densities differ among conservation action planning (CAP) areas over time? Our general prediction was that chimpanzee populations and their suitable habitat are declining over time and space. In general, our results will provide compelling arguments for practitioners and policymakers regarding the importance of conserving this stronghold of the eastern chimpanzee through appropriate and adaptive management actions that need to be underpinned by long‐term monitoring efforts.

## METHODS

### Study area

**FIGURE 1:**
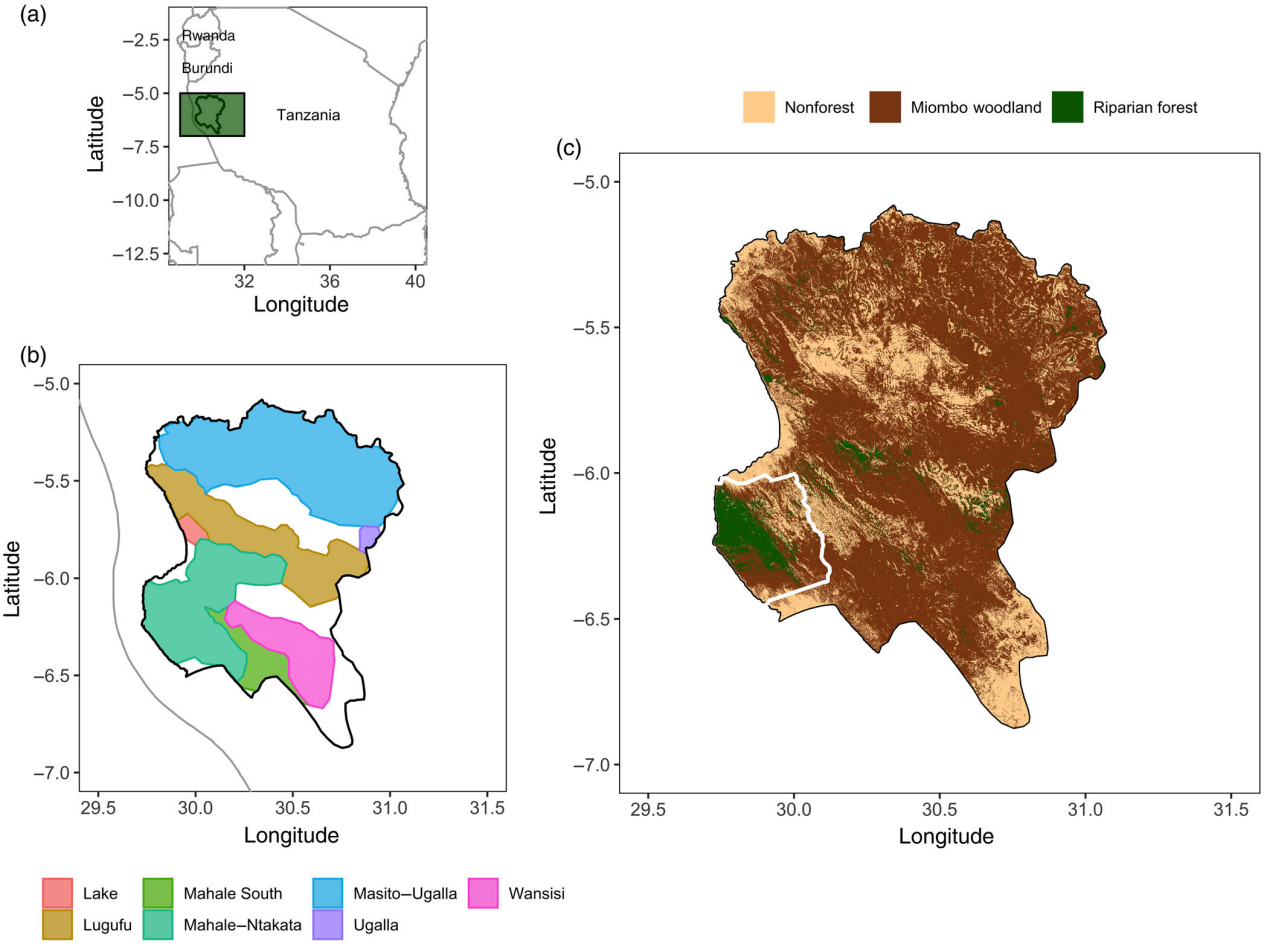
Location of (a) the Greater Mahale Ecosystem (GME) in western Tanzania, (b) chimpanzee conservation action plan (CAP) core ranges and corridors across the GME, and (c) main vegetation types across the GME and the Mahale Mountains National Park (MMNP, white).

The GME covers ~18,000 km^2^ in western Tanzania, and is characterized by steep mountains and flat plateaus, dominated by miombo woodland (71%, deciduous forests dominated by *Brachystegia* and *Julbernardia* [Fabaceae] species) with small patches of nonforest (23%; e.g., bare lands, grasslands, shrubs, cultivated fields, and human settlements) and riparian forest (6%, evergreen and dry forests) (Figure 1; Collins & McGrew, 1988; Kano, 1971; Ogawa et al., 2006; Yoshikawa et al., 2008). The GME is bounded by two major rivers, the Malagarasi in the north and the Ugalla in the east, by Katavi National Park in the south, and by Lake Tanganyika in the west. The climate is characterized by an annual temperature ranging from 11 to 38°C and annual precipitation varies between 900 and 2100 mm, with a wet season from November to April and a dry season from May to October (Collins & McGrew, 1988; Ogawa et al., 2006; Piel et al., 2017).

The Greater Mahale Ecosystem Research and Conservation Project (GMERC, https://www.gmerc.org/) has been coordinating and conducting chimpanzee surveys since 2005 across the GME in collaboration with the Jane Goodall Institute, The Nature Conservancy, Frankfurt Zoological Society, Tanzania Wildlife Research Institute (TAWIRI), Tanzania National Parks (TANAPA) and district and government partners. In collaboration with these institutions and local communities, the Jane Goodall Institute has been working on community‐led conservation projects across the Greater Gombe Ecosystem and GME since 1994 (Pintea et al., 2021; Pintea & Bean, 2022). As a result, district and village land‐use plans that cover nearly 6500 km^2^ have expanded new protected areas, of which 80% are local authority forest reserves managed at the district level and 20% of a variety of village forest reserves (e.g., private forests, woodlots, wildlife reserves) managed by local communities (Pintea et al., 2021; Wilson et al., 2020). Similarly, The Nature Conservancy (https://www.nature.org/en‐us/about‐us/where‐we‐work/africa/stories‐in‐africa/tuungane‐project) has established more than 20 Beach Management Units along Lake Tanganyika in the GME to improve fishing yields and take pressure off surrounding forests that may otherwise be converted to agriculture, and support livelihoods. The Frankfurt Zoological Society has pioneered a General Management Plan for Mahale Mountains National Park (MMNP), working to empower communities to protect land adjacent to the Park boundaries (Andres‐Bruemmer et al., 2021). Finally, Carbon Tanzania has successfully implemented REDD projects targeting the Ntakata Forest, perhaps the single most important refuge for chimpanzees in the GME outside of MMNP (Dickson et al., 2020). In protecting more than 2000 km^2^, they have raised almost US$750,000 for eight communities that patrol, monitor, and safeguard this critical area. Across Tanzania, chimpanzee range under some form of protection has increased from 9% in 2005 to 43% in 2019 (Wilson et al., 2020). Moreover, chimpanzee conservation priority areas have been identified as part of CAP processes (Figure 1) (TAWIRI, 2018).

### Chimpanzee nest surveys

Chimpanzees are elusive and occur at low densities in the GME (Moyer et al., 2006; Ogawa et al., 2013; Piel et al., 2015; Yoshikawa et al., 2008), and therefore nest counts were the most efficient means of establishing chimpanzee presence, estimating densities and monitoring their population trends (Buckland et al., 2010; Kuehl et al., 2008; Plumptre & Reynolds, 1996). We used nest data collected with the standing‐crop nest count method, which consists of documenting all nests that are observed from line transects, and measuring the perpendicular distance from the nest to the transect line (Plumptre & Reynolds, 1996). We only included nests classified according to the age categories defined by Plumptre and Reynolds (1997), in which the longevity of nests is considered until the decomposition of leaves (Stewart et al., 2011; Zamma & Makelele, 2012).

We used data from nest surveys conducted between 2010 and 2020, except 2016 when no surveys were conducted, using line‐transect distance sampling (Appendix S1: Figure S1). Survey design followed one of three different approaches. In MMNP, we followed a systematic random design (102 transects in 13 sites, length 0.4–1.0 km; Chitayat et al., 2021). In the Masito–Ugalla Ecosystem, we walked line transects (77 transects in 12 sites, length 0.9–7.3 km) that originated from a central point and followed cardinal directions. Finally, where chimpanzees were known to be present, we established parallel transects (454 transects in 16 sites, length 0.9–7.0 km), following a randomly selected bearing, and spaced at 0.5 km across the area (Moyer et al., 2006; Piel et al., 2015; Piel & Stewart, 2014). We split all transects into segments based on the shortest transect length (0.4 km) using the *dshm* package version 0.1.0 in R (Franchini, 2018).

All research complied with protocols approved by the TAWIRI and adhered to the legal requirements of Tanzania.

### Predictor variables

We selected a set of predictors for density based on their known importance for chimpanzee distribution across the GME (Bonnin et al., 2020; Chitayat et al., 2021; Hernandez‐Aguilar et al., 2013; Jantz et al., 2016; Nishida & Shigeo, 1983; Ogawa et al., 2013; Piel et al., 2017), while guaranteeing data availability for the time series considered at a high resolution, and minimizing correlation among variables (Appendix S1: Figure S2, Table S1). We compiled annual precipitation and annual temperature range from Worldclim (please refer to Appendix S1: Table S1; Hijmans et al., 2005), based on its influence on chimpanzee distribution (Carvalho et al., 2021). We extracted human population density from a high‐resolution human population data set (Lloyd et al., 2019) as a proxy of human activities affecting the distribution of chimpanzees (TAWIRI, 2018). We further used tree cover data derived from remote sensing data (Bonnin et al., 2020; Appendix S1: Table S1). Three categories were considered based on the two major macrohabitats used by chimpanzees in Tanzania: forest, tree cover >65%; woodland, tree cover >25% to <65%; and nonforest, <25% tree cover. To obtain vegetation types between 2010 and 2019, we used tree cover loss data sets from Hansen et al. (2013) to reclassify forested areas into nonforest.

We compiled elevation from the Shuttle Radar Topographic Mission (SRTM) Digital Elevation Model (DEM) data set. Elevation is a potentially important predictor of chimpanzee distribution, as it determines nesting site location and food resource distribution (Jantz et al., 2016; Ogawa et al., 2013). We derived slope from this data set given that steep slopes might provide protection from predators and thermoregulatory benefit, and offer a greater concentration of trees suitable for nesting (Hernandez‐Aguilar, 2009). Steep slopes were defined as those >20° (Jantz et al., 2016; Moyer et al., 2006). Layers representing distances to steep slopes, as well as to forests were obtained by calculating Euclidean distances in ArcMap version 10.7 (Esri, 2011).

We assessed collinearity by calculating the variance inflation factor (VIF) in the R package *usdm* (Naimi et al., 2014) and eliminated predictors with a VIF >3. After the exclusion of annual precipitation, which had the highest VIF (6.6) and was strongly correlated with elevation, we considered all remaining predictors for the modeling as they had VIFs <2 (Appendix S1: Figure S3, Table S2).

### Density surface modeling

We used DSMs to model nest counts corrected for detectability as a function of predictor variables, and subsequently to predict chimpanzee abundances and densities over time and space. We implemented the DSMs in a two‐stage approach by (1) fitting a detection function to the distance data to account for detectability to estimate abundances on each transect segment with a Horvitz–Thompson‐like estimator (Borchers et al., 1998), and (2) implemented a GAM (Wood, 2017) to estimate abundance per transect segment and spatially predict it over a larger, different area than was initially sampled (Miller et al., 2013). We implemented this approach using the packages *Distance* version 0.9.8 (Miller et al., 2019) and *dsm* version 2.2.7 in R (Miller et al., 2013).

#### Stage 1: Detection probability estimation

We first explored the distance data in histograms, considering different truncation distances and fitting a half‐normal model without adjustment terms to visualize the shape of the detection function and select the best truncation distance based on the rule of thumb = 0.15 (Buckland et al., 2015). Subsequently, we considered candidate detection function models either including vegetation type as covariate (Marques et al., 2007) or not (Buckland et al., 2015) such as half‐normal, hazard‐rate and uniform key functions with at most cosine adjustment terms of order five. We evaluated the goodness of fit of the models based on Cramer–von Mises and Kolmogorov–Smirnov tests (Buckland et al., 2015) and selected the best model using Akaike's Information Criterion (AIC).

#### Stage 2: Model‐based density estimation

We fitted the following GAM to model nest counts as a function of smooth functions of predictors:

$$\begin{array}{ll} \log\left(n_{\mathrm{it}}\right)\sim & f_{1}\left(\text{annual temperature range}\right)\\ & +\;f_{2}\left(\text{distance to steep slopes}\right)+\;f_{3}\left(\text{elevation}\right)\\ & +f_{4}\left(\text{distance to forests}\right)\\ & +\;f_{5}\left(\text{vegetation type},k=2\right)+\;f_{6}\left({\text{year}}_{i},k=7\right)\\ & +\;f_{7}\left({\text{longitude}}_{t},{\text{latitude}}_{t}\right)\\ & +\;f_{8}\left({\text{longitude}}_{t},{\text{year}}_{i}\right)+\;f_{9}\left({\text{latitude}}_{t},{\text{year}}_{i}\right)\\ & +\;f_{10}\left({\text{longitude}}_{t},{\text{latitude}}_{t},{\text{year}}_{i}\right)\\ & +\log\left(\text{human population density}\right)\\ & +\;\text{offset}\left(\text{detectability}\right) \end{array}$$

where *n*_*it*_ is the nest count in the *i*‐th year at the *t*‐th transect segment, *f*_1–10_ are the smooth terms, and *k* represents the largest complexity for each smooth term. Smoothers (*f*_1–10_) are represented by random effects and were modeled as thin plate regression splines (Wood, 2003, 2017), which are isotropic, that is, there is only one parameter controlling the smoothness in both directions. Thin plate splines are appropriate when using UTM coordinates, which are isotropic as well (Camp et al., 2020). For the interaction between year and location (*f*_7–10_), we used a tensor product as these predictors are anisotropic given that their units are in different scales (Wood, 2003, 2017). Human population density was included as a linear term given the high difference between the effective and reference degrees of freedom. An offset for each transect segment that includes the estimated detectability was also included.

We considered two distribution families for the nest counts, negative binomial, and Tweedie, both with a log‐link function. We selected the best model based on the lowest AIC (∆AIC <2) and highest deviance explained, and then validated the best model by inspecting deviance residuals in quantile–quantile (QQ) plots and checking for normal distribution and constant variance (Wood, 2017). We then validated model performance using a 10‐fold crossvalidation with a random training–testing split. We obtained the average as the final estimate and compare the predicted versus, observed nest abundances using the following evaluation metrics: root mean square error (RMSE), mean absolute error (MAE), and R‐squared (*R* ^2^). RMSE was used to measure the average squared difference between the predicted values and the observed values, and MAE was used to reflect the actual situation of the predicted value error. *R* ^2^ is a statistic that measures the goodness of fit and corresponds to the squared correlation between the predicted values and the observed values. Given that the model for detection function estimation included a covariate, we used a GAM uncertainty estimation and combined it with the detection function uncertainty via the delta method (Seber, 1973). For this, we used the *dsm.var.gam* function to obtain the total uncertainty in the spatial model for each vegetation type per year.

### Estimation of chimpanzee densities

We predicted weaned chimpanzee densities/km^2^ by

(1) $$\text{Dchimps}\;=\;\frac{\text{Dnests}}{\mathrm{tr}}\;$$

where *t* corresponds to the nest production rate and *r* is the nest decay rate. We used 1.09 nests/day for nest production rate, as described previously (Plumptre & Reynolds, 1996, 1997). Nest decay varies across the GME, and is particularly affected by season, vegetation type, and proximity to Lake Tanganyika (Stewart et al., 2011; Zamma & Makelele, 2012). Therefore, we divided the GME into two regions—lakeshore and inland—and considered nest decays (average of wet and dry seasons) by vegetation type, applying nest decay rates from Zamma and Makelele (2012) to the lakeshore and the only available dry habitat nest decay rates from Stewart et al. (2011) to the rest of the GME (Appendix S1: Table S3).

Considering a grid of 1 × 1 km, we predicted chimpanzee densities and abundances by vegetation type over time, as well as the corresponding confidence intervals (CIs). Additionally, we represented the uncertainty in these estimates in the form of standard deviation (SD) maps given the expected variation of chimpanzee densities across the GME. We finally extracted the predicted chimpanzee densities (mean and respective SD) per year for each CAP area.

## RESULTS

In total, 1518 km of transects (*N* = 633) were walked between 2010 and 2020. We placed most transects in woodland (*N* = 336), followed by nonforest (*N* = 243) and forest (*N* = 54). Sampling effort varied by year (mean = 152, SD = 134, range 23–422 km), and consequently there was large variation in the number of nests recorded (mean = 423, SD = 470, range 128–1691; Appendix S1: Figure S4). We recorded a total of 4232 nests, mostly in woodland (59%), followed by forest (36%) and nonforest (5%).

### Stage 1: Detection probability estimation

We set the truncation distance at 50 m, yielding 4057 nests from 509 transect segments (Appendix S1: Figures S4–S6, Table S4). We detected the most nests in woodland (56%) and forest (37%), and the fewest nests in nonforest (5%) (Appendix S1: Figure S5). The model selected for inference was the half‐normal with vegetation type as covariate and the average detection probability obtained was 0.47 (CV [coefficient of variation] = 0.01) (Appendix S1: Figure S7, Table S5).

### Stage 2: Model‐based density estimation

By comparing the AIC scores and residual QQ plots, we found that a model with the negative binomial distribution with an estimated overdispersion parameter of 0.15% and 43.4% of deviance explained provided the best fit for the nest data (Appendix S1: Figures S8, S9; Tables S6, S7). The ability of this model to predict nest abundance was good (RMSE = 1.35, MAE = 0.53, *R* ^2^ = 0.27; Appendix S1: Figure S10). The location of the transect segments and survey year, distance to slopes, distance to forests and vegetation type were important predictors of chimpanzee nests (Table 1). Moreover, human populations negatively influenced the location of the chimpanzee nests (Table 1). Plots of the smooth terms suggest that more nests were found close (<500 m) to slopes and to forests and in woodlands (Appendix S1: Figure S11). The CV from the GAM ranged from 0.19 to 0.37 across vegetation types over time.

**TABLE 1:** Results of the best‐fit generalized additive model (GAM) with linear effects (linear predictors) and smooth terms (nonlinear predictors) assessing the relationship between chimpanzee nests and environmental predictors.

Predictors	β	SE	*t*	edf	rf	χ^2^	*p*‐value
*Linear effects* ^a^							
Intercept	−12.66	0.60	−21.26	…	…	…	**<0.001**
Human population	−0.37	0.18	−2.10	…	…	…	**<0.05**
*Smooth terms*							
Year				0.93e−2	6	0.90e−02	0.32
Longitude, latitude				25.60	29	234.35	**<0.001**
Longitude, year				0.22e−2	16	0.01	0.54
Latitude, year				5.53	16	16.94	**<0.01**
Longitude, latitude, year				0.14e−02	64	0.01	0.93
Distance to slopes				4.76	9	241.21	**<0.001**
Elevation				0.12	9	0.12	0.34
Distance to forests				2.67	9	86.32	**<0.001**
Vegetation type				0.90	2	9.37	**<0.01**

*Note*: Values in bold face indicate significant differences.

Abbreviations: β, parameter estimates; *edf*, effective degrees of freedom; *rf*, reference degrees of freedom; *SE*, standard error; *t*: *t*‐value; χ^*2*^, χ^*2*^ statistic.

^a^ *R* ^2^ (adj.) = 0.04, Scale est. = 1, *n* = 4057.

**FIGURE 2:**
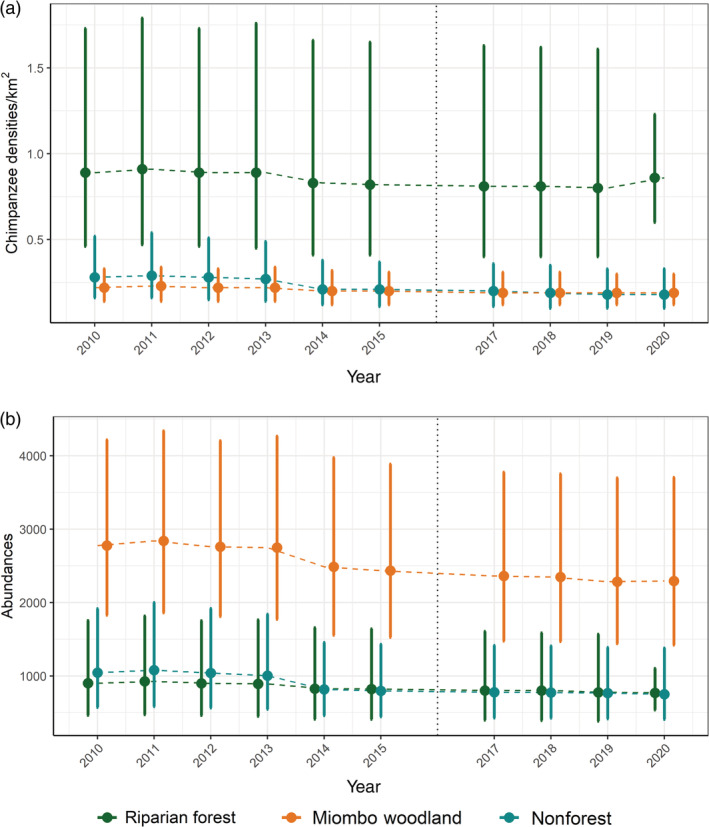
Temporal variation in estimates (mean and 95% confidence intervals) of (a) chimpanzee densities and (b) abundances for each vegetation type. No surveys were conducted in 2016.

On average, we obtained an annual population decline of 2.41% across the GME, including a decline of 3.45% in nonforests, 2.05% in woodlands, and 1.72% in forests per year. The highest chimpanzee density was obtained for the least available vegetation type, that is, forest, for which we estimated 0.86 chimpanzees/km^2^ in 2020 (95% CI 0.60–1.23), followed by 0.19 chimpanzees/km^2^ in woodland (95% CI 0.12–0.30) and 0.18 chimpanzees/km^2^ in nonforest (95% CI 0.10–0.33) (Figure 2). Chimpanzees need access to a mosaic of both forest and woodland, however, because of its large extent, woodlands support most of the chimpanzee population (2294 chimpanzees, 95% CI 1420–3707; as of 2020), with only half of that figure estimated for forest (769 chimpanzees, 95% CI 536–1103) and for nonforest (750 chimpanzees, 95% CI 408–1381).

Our model predicted a decrease in both density and abundance across the GME over time (Figure 2). A slight increase in both density and abundance was predicted between 2010 and 2011, declining from 2012 onwards in all vegetation types. Importantly, habitat trends were more consistent over time, forests annually decreased 1.36% and woodlands 0.32%, with a total reduction of 13.60% and 3.25% by 2020, respectively (Appendix S1: Figure S2). In contrast, an annual increase of 1.35% was obtained in nonforests, with a total increase of 13.45% by 2020.

**FIGURE 3:**
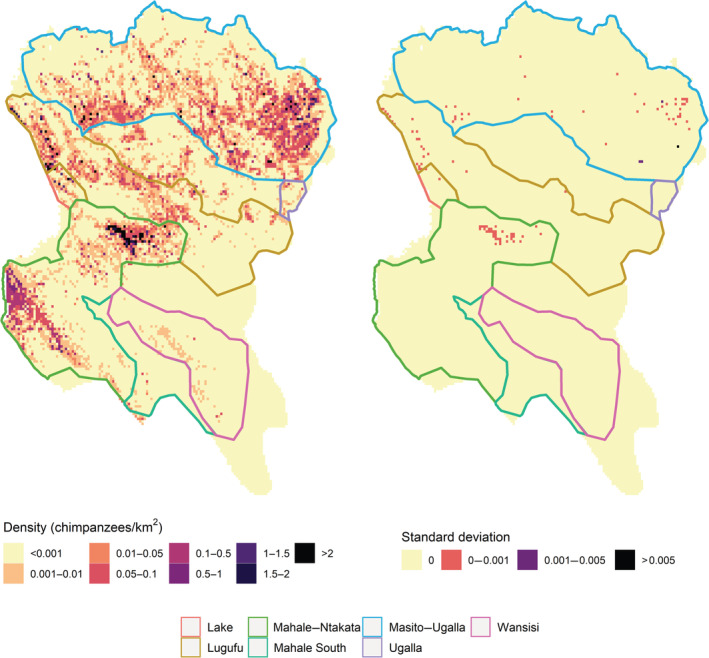
Predicted chimpanzee densities (number of individuals/km^2^), and respective uncertainty (standard deviation) in these estimates across the Greater Mahale Ecosystem (GME) for 2020. The boundaries of conservation action plan (CAP) areas are also shown.

**FIGURE 4:**
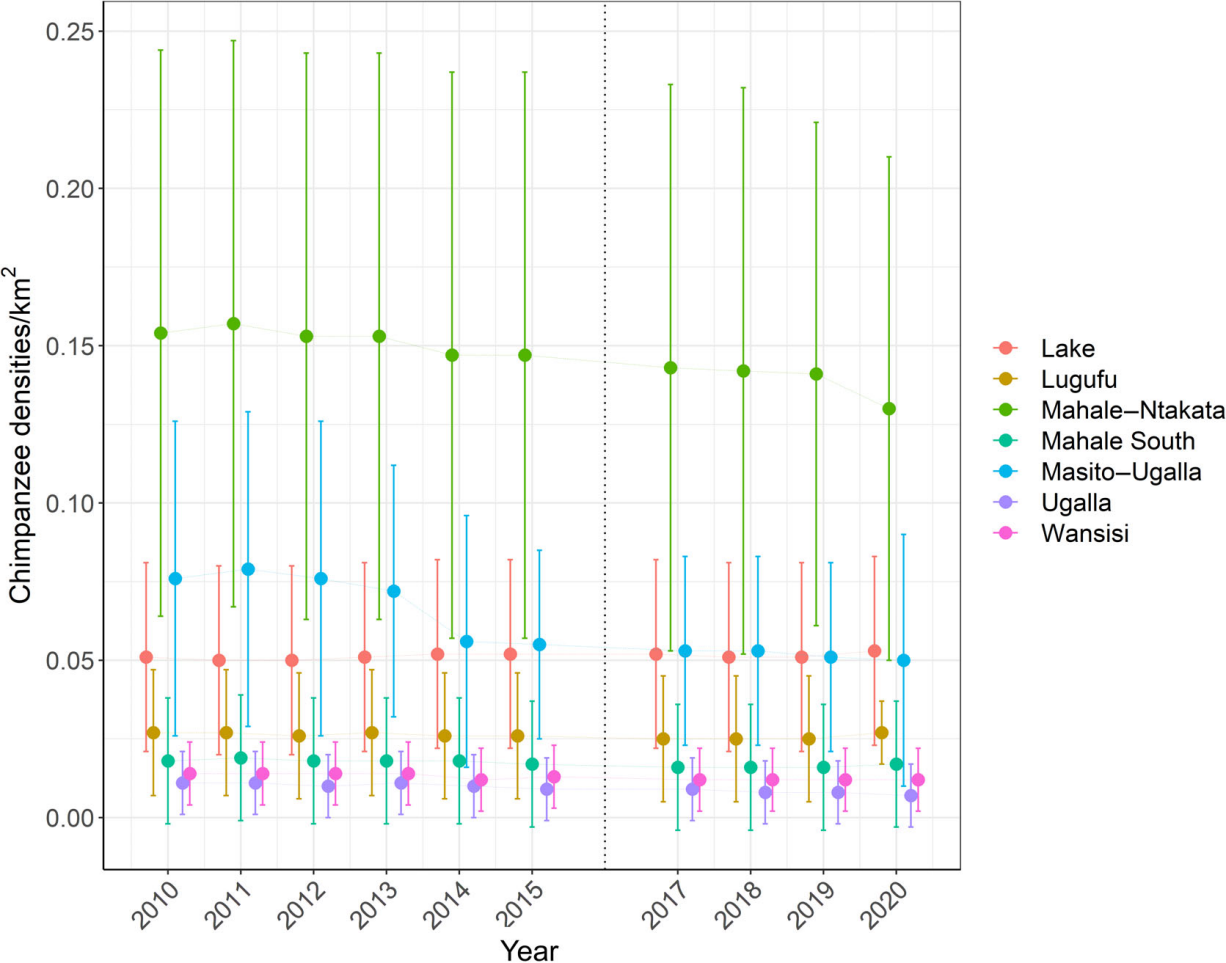
Temporal variation in the predicted chimpanzee densities (mean and respective standard deviation) for each conservation action plan (CAP) area. No surveys were conducted in 2016.

Our model predicted the highest density estimate for Mahale and Ntakata regions (mean = 0.13 chimpanzees/km^2^, SD = 0.08; as of 2020) and the lowest density estimate for Ugalla (mean = 0.01 chimpanzees/km^2^, SD = 0.01; as of 2020) (Figures 3, 4). A slight increase in densities was obtained in Lake between 2012 and 2017, declining between 2018 and 2019 and increasing again in 2019, and in Mahale South, Mahale–Ntakata, and Masito–Ugalla between 2010 and 2011, declining from 2012 onwards. Chimpanzee densities in Lake, Ugalla, and Wansisi were predicted to be stable over time, in contrast with the decreasing trend obtained for Masito–Ugalla between 2010 and 2020 (Figure 4). Uncertainty in density estimates was highest in Ntakata, Lugufu and Masito–Ugalla regions (Figure 3).

## DISCUSSION

Across their range, chimpanzees are under extreme pressure from habitat loss and fragmentation, disease, and poaching (Heinicke et al., 2019; Junker et al., 2012; Plumptre et al., 2010). Detailed information about their distribution and population size in combination with monitoring populations over time and space is essential to mitigate the negative synergistic effects of these anthropogenic threats driving declines in abundance and, in some cases, population extinctions. This information reveals not only population status and threat trends, but relevant information about the effectiveness of conservation programs and management strategies (Kuehl et al., 2008).

By integrating a 10‐year time series data set of chimpanzee surveys across the GME with relevant environmental information, our DSM, in line with our initial hypothesis, predicted a decline in chimpanzee populations over time and space, in agreement with previous studies (Piel et al., 2015; Plumptre et al., 2010; Yoshikawa et al., 2008). We further found that GME chimpanzee populations are shrinking, on average, at an annual rate of 2.41%, which is within the range 2%–7% of annual population declines reported globally for great apes (Kuehl et al., 2017; Plumptre et al., 2016; Strindberg et al., 2018; Wich et al., 2016). These population declines were accompanied by ecosystem‐wide declines of 1.36% and 0.32% per year for the two key vegetation types on which chimpanzees rely, forest and woodland, respectively, a figure that falls within the same rate reported for forest area loss globally (Winkler et al., 2021). Based on our model, we estimate there to be 3813 chimpanzees as of 2020 across the GME, which is within the range of previous estimates using design‐based inferences. Moyer et al. (2006) provided the first chimpanzee population status for the GME by conducting line‐transect distance sampling along nonrandomly selected line transects only placed within forest and woodland areas suitable as nesting sites (total effort = 203 km). These were then extrapolated to habitats predicted to be suitable for chimpanzees using species distribution modeling and satellite image, reporting a total of 2620 individuals. By following the same approach, Ogawa et al. (2013) conducted nest surveys along nonrandomly selected line transects placed in paths across suitable (total effort = 1026 km) and nonsuitable (total effort = 46 km) habitats for chimpanzees outside protected areas. Ogawa and colleagues then added previous estimates for MMNP (Moyer et al., 2006) as well as areas outside the Park (Yoshikawa et al., 2008), and suggested a far smaller number (*N* = 1200) of chimpanzees for the GME. However, these studies differed in key aspects. Moyer et al. (2006) sampled more forests and considered different proportions of vegetation types to extrapolate abundances using a decay rate of 97 days (to leaf decomposition), whereas Ogawa et al. (2013) used decay rates three times higher of 260 days (to complete disappearance of the nest). These methodological differences may in part explain the disparate abundance estimates from these studies. In comparison, our model‐based approach (1) integrated nest data from both a systematic random design of line transects across the Mahale region (whereas largest patches of forests can be found withing the MMNP) and nonrandomly selected line transects across the remaining CAP areas (total effort = 1518 km), (2) considered major vegetation types relevant to chimpanzees across the GME extracted from satellite image analysis, (3) took into account different nest decays across different regions and vegetation types (Appendix S1: Table S3), and (4) investigated the relationship between nest abundance and environmental predictors.

We estimated a mean density of 0.86 chimpanzees/km^2^ in forest (95% CI 0.60–1.23), followed by 0.19 chimpanzees/km^2^ in woodland (95% CI 0.12–0.30) and 0.18 chimpanzees/km^2^ in nonforest (95% CI 0.10–0.33). The sum of these estimates (1.2) is similar to the previous estimate of 1.3 individuals/km^2^ (95% CI 0.4–3.2) reported for only forest and woodland areas suitable as nesting sites (Moyer et al., 2006), these are areas that in fact represent one‐tenth of the total area of the GME (2015 vs. 18,000 km^2^, in Moyer's and this study, respectively). We also obtained the highest densities in forests, very close to the figure reported by Moyer et al. (2006) (0.86 vs. 1.4, respectively). During the last decade, chimpanzee densities slightly decreased in Mahale, Ntakata, and Masito–Ugalla, except Lake, Mahale South, Ugalla, and Wansisi, where they were estimated to remain constant over time (Figures 3 and 4). Importantly, our estimates were in line with previous densities in Masito–Ugalla (Moyer et al., 2006; Piel et al., 2015; Piel & Stewart, 2014; Suzuki, 1969; Yoshikawa et al., 2008), in MMNP (Chitayat et al., 2021; Moyer et al., 2006), in Ntakata and Wansisi (Ogawa et al., 2006; Yoshikawa et al., 2008), and for all of the southern GME (Piel & Stewart, 2014).

As previously reported for the GME (Hernandez‐Aguilar, 2009; Hernandez‐Aguilar et al., 2013; Moyer et al., 2006; Piel et al., 2015), as well as for sites at the western margins of chimpanzee distribution in Africa (Carvalho et al., 2013; Pruetz et al., 2002), more nests are proportionally detected in or near forests than in woodlands, given that the former is proportionately underrepresented compared with the latter. Forests provide suitable conditions for nesting and feeding and might also offer protection from predators, or thermoregulatory benefits (Hernandez‐Aguilar, 2009; Nishida & Shigeo, 1983; Ogawa et al., 2013; Piel et al., 2017; Stewart et al., 2011). These forests are distributed in thin strips surrounded by woodlands where more than half of chimpanzee populations occur at low densities and also find suitable conditions for feeding, nesting and traveling between forest patches (Hernandez‐Aguilar, 2009). Therefore, it is imperative to conserve both vegetation types when devising effective habitat connectivity policies to ensure gene flow among chimpanzee populations and their long‐term viability (Bonnin et al., 2020).

## LIMITATIONS OF THE NEST SURVEYS

More nests than expected were detected in the first data interval (Appendix S1: Figure S6), suggesting that upward bias may have been introduced through systematic errors in the measurement of distances in nest data collected close to the transect by rounding the true distances to zero.

Nest decay is affected by season, vegetation type, and here, the distance from Lake Tanganyika (Stewart et al., 2011; Zamma & Makelele, 2012). While a strict delineation based on weather patterns is difficult to achieve, we aggregated areas more likely to experience similar conditions to those where the decay rates were measured. However, the influence of certain covariates on nest decay may differ between the time and location where nest decay and chimpanzee surveys were performed, leading to bias (Marques et al., 2013). Therefore, we recommend that future surveys collect information on nest decay at the same time or under the same conditions as those during chimpanzee surveys for realistic estimates of abundances (Marques et al., 2013), by following the marked nest count method (Plumptre & Reynolds, 1996).

## CONCLUSION

Reliable estimates of chimpanzee distribution and population size, in combination with threats driving population changes over time and space, are central to programs and policies designed to effectively conserve chimpanzees. DSMs provide reliable estimates to better support conservation decisions as they inform about the spatio‐temporal distribution of species abundance and density inferred from a spatial model containing relevant environmental predictors (Buckland et al., 2000; Hedley & Buckland, 2004; Miller et al., 2013). Our results corroborate other recent studies showing that chimpanzees and their habitats are declining (Junker et al., 2012; Kuehl et al., 2017; Plumptre et al., 2016; Strindberg et al., 2018). However, our estimated annual decline of chimpanzees is lower than that reported for western chimpanzees (2.41% and 5.96%, respectively) (Kuehl et al., 2017). This less severe decline is likely to be the result of years of conservation interventions and cultural values across western Tanzania.

Conservation organizations and research stations have been conducting chimpanzee surveys in the region since 1960 and, more recently, developing spatially explicit conservation action plans and village, district, and regional land‐use plans in collaboration with government authorities and local communities (Pintea et al., 2021; TAWIRI, 2018; Wilson et al., 2020). As a result, chimpanzee range under some form of protection increased in Tanzania from 9% in 2005 to 43% in 2019 through a network of national parks, village, district and central forest reserves (Wilson et al., 2020). The cultural values of local communities such as the Bende/Tongwe people are also likely to play an important role in protecting the GME and its wildlife (Infield et al., 2018; Jumanne, 2014). These tribes have a deep spiritual connection with their rivers, mountains, and forests and do not consume chimpanzees due to their resemblance to humans (Jumanne, 2014; Nakamura, 2015). In contrast, poaching is identified as one of the highest threats to chimpanzees across West Africa along with habitat loss (UCN SSC, 2020). Deforestation is also more widespread in this part of Africa with large‐scale industrial farming representing the major driver of deforestation (Ruf et al., 2014), whereas deforestation in Tanzania is largely driven by small‐scale subsistence agriculture (Doggart et al., 2020). Although chimpanzees in the GME are declining at a lower rate than in other parts of their geographic range, small declines will eventually accumulate over time causing substantial losses. Therefore, we recommend long‐term monitoring programs relying on a systematic random design to effectively guide and allocate conservation actions. Importantly, inclusion of predictors that capture key threats, such as disease, directly contributing to the decline of chimpanzee populations, and use of a model‐based approach should be considered in future work.

Despite the GME being relatively remote, a recent diverse mixture of cultural groups have moved into this region, both from natural growth and immigration, relying almost entirely on natural resources from inside and outside protected areas and following a more pastoralist and agriculturalist lifestyle (Kideghesho, 2016; Whitaker, 2002). Consequently, several human activities such as agriculture, infrastructure and settlement development, unsustainable extraction of timber and firewood and charcoal production, livestock keeping, and mining are now acting as major drivers of habitat loss and fragmentation in this ecosystem and, therefore, leading to the loss of suitable areas for chimpanzees (Doggart et al., 2020; Ogawa et al., 2006; Piel et al., 2015; Plumptre et al., 2010).

Key core ranges and corridors to maintain connectivity among chimpanzee populations across the GME and to ensure gene flow among populations and their viability into the future have been identified (Bonnin et al., 2020; TAWIRI, 2018). Most of these suitable areas occurs outside MMNP and covers the existing network of village and district forest reserves that struggle to stop illegal expansion of settlements, agriculture and livestock in those areas, and future loss is predicted by 2027 (Dickson et al., 2020). Therefore, to avert chimpanzee extinctions in the long term and increase protection of important areas for connectivity between suitable habitats, it will be critical to (1) continue building village and district government capacity and provide additional resources and tools to more effectively enforce their forest reserves, ensure objective monitoring and assessments of human activities, and share these data with local decision makers; (2) identify hotspots of forest loss and future risks in the coming years by integrating multiscale drivers of deforestation across the GME as predictors in a modeling approach (Cushman et al., 2017); in combination with our findings, (3) prioritize monitoring and enforcement of forest reserves through initiatives such as REDD+ carbon forest projects (Dickson et al., 2020) and community‐led conservation strategies (Pintea et al., 2021; Pintea & Bean, 2022). Village and district land‐use plans should be used as a legal framework to direct new settlements, agriculture and livestock activities in areas where the impact on chimpanzees would be minimal; and (4) mitigate climate change impacts on forest productivity and distribution (John et al., 2020) by incorporating this driver into land‐use planning and propose mitigation measures for suitable habitats across this critical ecosystem for chimpanzees and other wildlife.

## CONFLICT OF INTEREST

The authors declare no conflict of interest.

## Supporting information


Appendix S1

## Data Availability

Data sets and R scripts (Carvalho et al., 2022) are available in Figshare at https://doi.org/10.6084/m9.figshare.19137509. Environmental data sets used here are cited with sources available in Appendix S1: Table S1.
