# Supplementary material for: Spatio‐temporal changes in chimpanzee density and abundance in the Greater Mahale Ecosystem, Tanzania
Source: Ecol Appl. 2022 Sep 30;32(8):e2715. doi: 10.1002/eap.2715 (PMC10078593; doi:10.1002/eap.2715)
Supplement: Supplementary file 1 — Appendix S1 [file EAP-32-0-s001.pdf]

Spatio-temporal changes in chimpanzee density and abundance in the Greater Mahale Ecosystem, Tanzania

APPENDIX S1

Joana S. Carvalho, Fiona A. Stewart, Tiago A. Marques, Noemie Bonnin, Lilian Pintea, Adrienne Chitayat, Rebecca Ingram, Richard J. Moore, Alex K. Piel

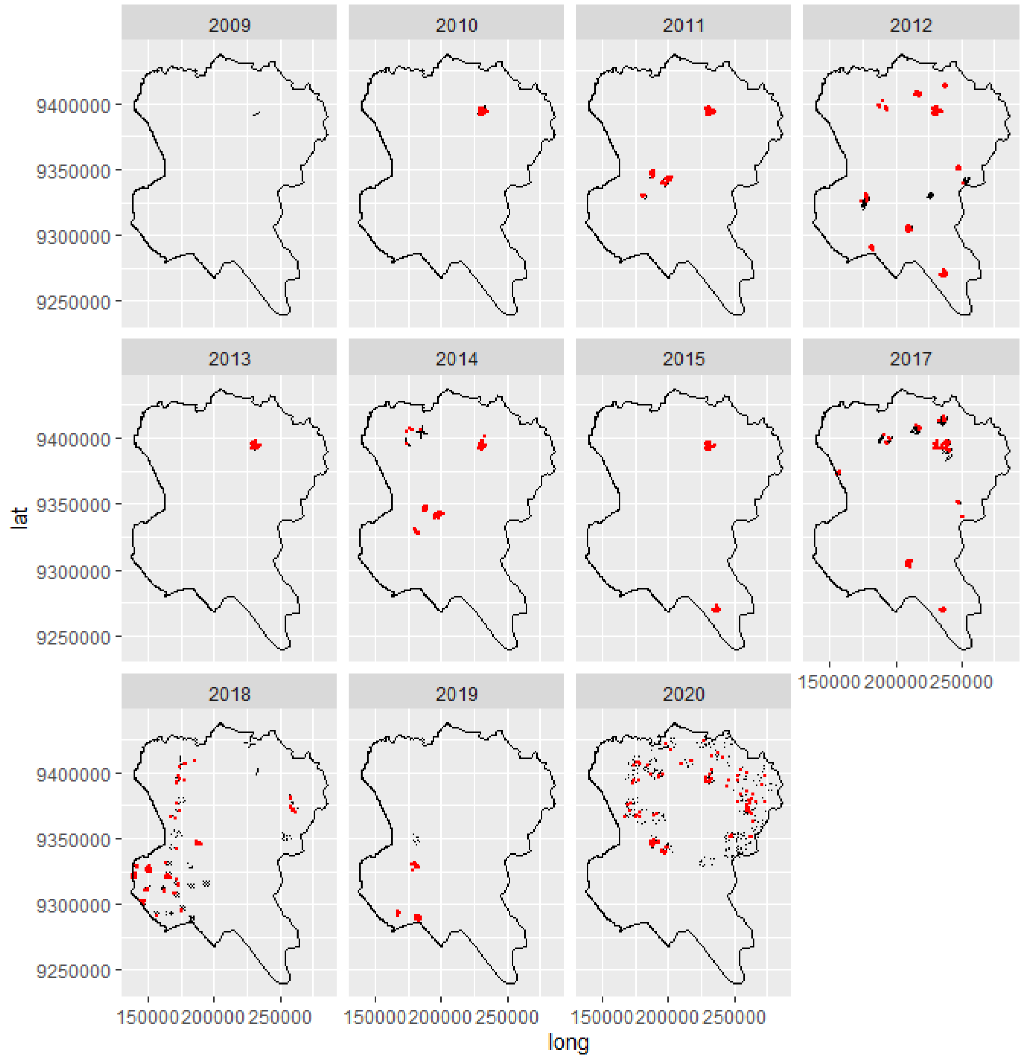

Figure S1. Location of the line transects (black lines) and chimpanzee nests (red dots) between 2010 and 2020, except 2016.

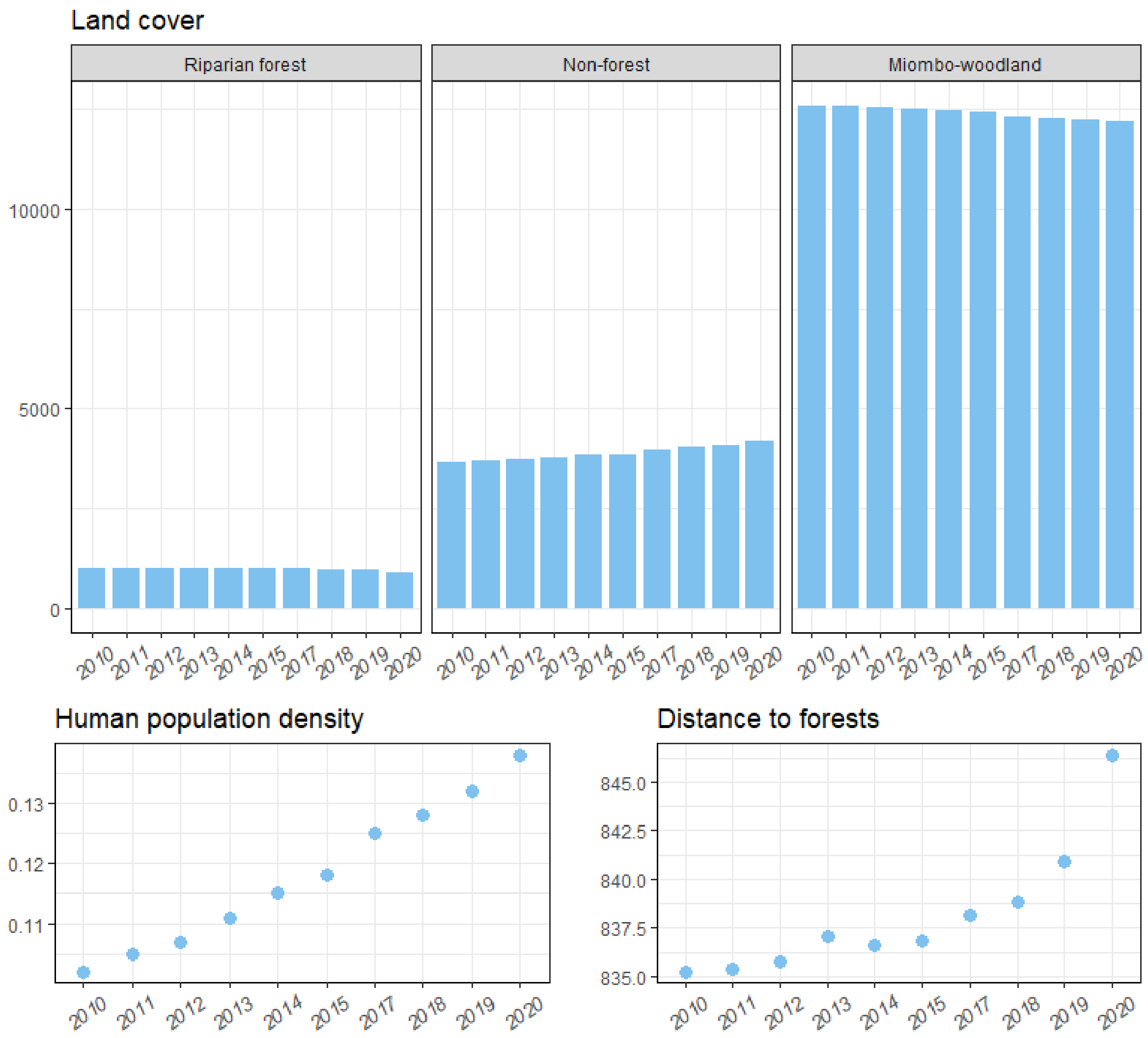

Figure S2. Changes in predictor variables (mean) across the GME used for predicting chimpanzee densities between 2010-2020, except 2016. Land cover (vegetation type) and human population density are in km^2, and distance to forests in km.

Table S1. List of the environmental predictors used in the modelling and their respective sources.

Type	Temporal Coverage	Resolution	Source
Vegetation type	2010-2020	30 m	**Jane Goodall Institute** Land cover data was derived from the Landsat-1 Multispectral Scanner (MSS, August 17, http://earthexplorer.usgs.gov at 60-meter resolution) and Landsat-7 Enhanced Thematic Mapper (ETM) and Landsat-8 satellite imagery (2000 images). Then, a tree cover layer was created at 30-m resolution using all Landsat ETM images for 2000 and adopting the methodology by Hansen et al. (2013).
Elevation and Slope	2000	30 m	https://earthexplorer.usgs.gov/
Annual precipitation, annual temperature range	2000	1 km	https://www.worldclim.org
Human population density	2010-2020	30 m	https://www.worldpop.org/geodata/listing?id=29

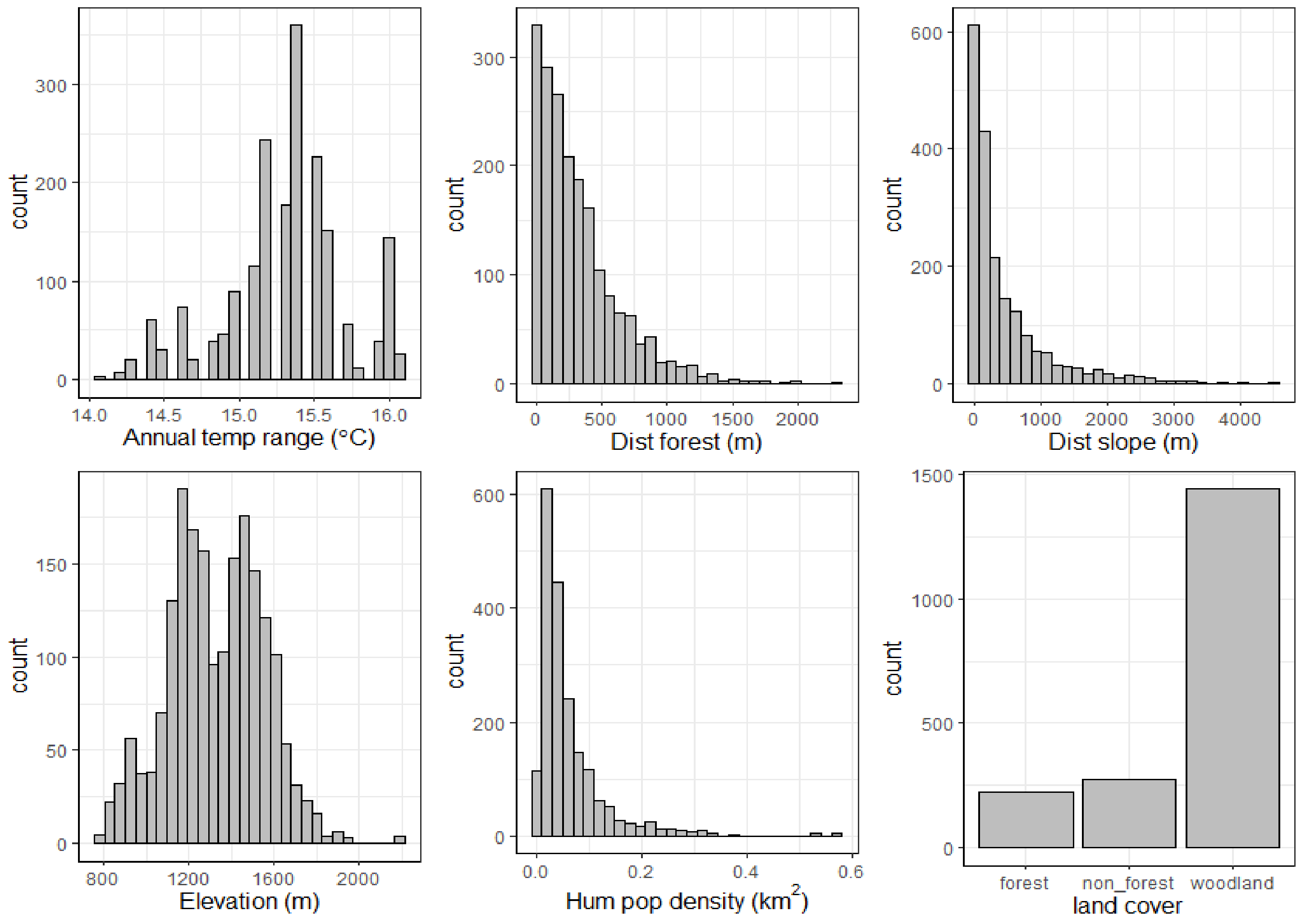

Figure S3. Histograms of the environmental predictors considered for the modelling.

Table S2. Results of the variance inflation factor (VIF) for each predictor.

Predictor	VIF
Elevation	1.23
Annual temperature range	1.18
Distance to forests	1.13
Human population density	1.12
Distance to slopes	1.09
Vegetation type	1.06

Table S3. Nest production and decay rates used in this study for lakeshore and inland regions to estimate densities by vegetation type.

Region	Vegetation type	Nest production	Nest decay
Lakeshore	Woodland	1.09	126*
	Forest	1.09	62.5*
	Non-forest	1.09	126*
Inland	Woodland	1.09	162.3**
	Forest	1.09	101.1**
	Non-forest	1.09	162.3**

*Average based on nest decays from Zamma and Makelele (2012); **average based on nest decay from Stewart et al. (2011). These nest decay rates were classified according to the age categories defined by Plumptre and Reynold (1997), in which the longevity of nests is considered until the decomposition of leaves.

Nest decay estimation

Nest decay varies across the GME, and is particularly affected by season, vegetation type and proximity to lake Tanganyika (Stewart, Piel, & McGrew, 2011; Zamma & Makelele, 2012). Faster nest decay rates were reported in two study sites, Kasoje and Miyako, located within the MMNP and close to the lakeshore (Zamma & Makelele, 2012), compared to Issa Valley, a study site located further inland (Stewart et al., 2011). This may be due to greater rainfall near the lakeshore (Collins & McGrew, 1988). Moreover, vegetation changes from predominantly forest on the west of the Mahale mountain range exposed to the lake to predominantly dry deciduous (miombo) woodland on the other side (Collins & McGrew, 1988). Thus, we divided the GME into two regions - lakeshore and inland - considering lakeshore to be within 6 km of the shoreline. This distance was based on the tallest peak found in the Mahale mountain range, Nkungwe Hill at 2462 m (Collins & McGrew, 1988), which is located directly to the east of the MMNP nest decay study locations surveyed by Zamma and Makelele (2012). Accordingly, we considered nest decays (average of rainy and dry seasons) by vegetation type, applying nest decay rates from Zamma and Makelele (2012) to the lakeshore and the only available dry habitat nest decay rates from Stewart et al. (2011) to the rest of the GME.

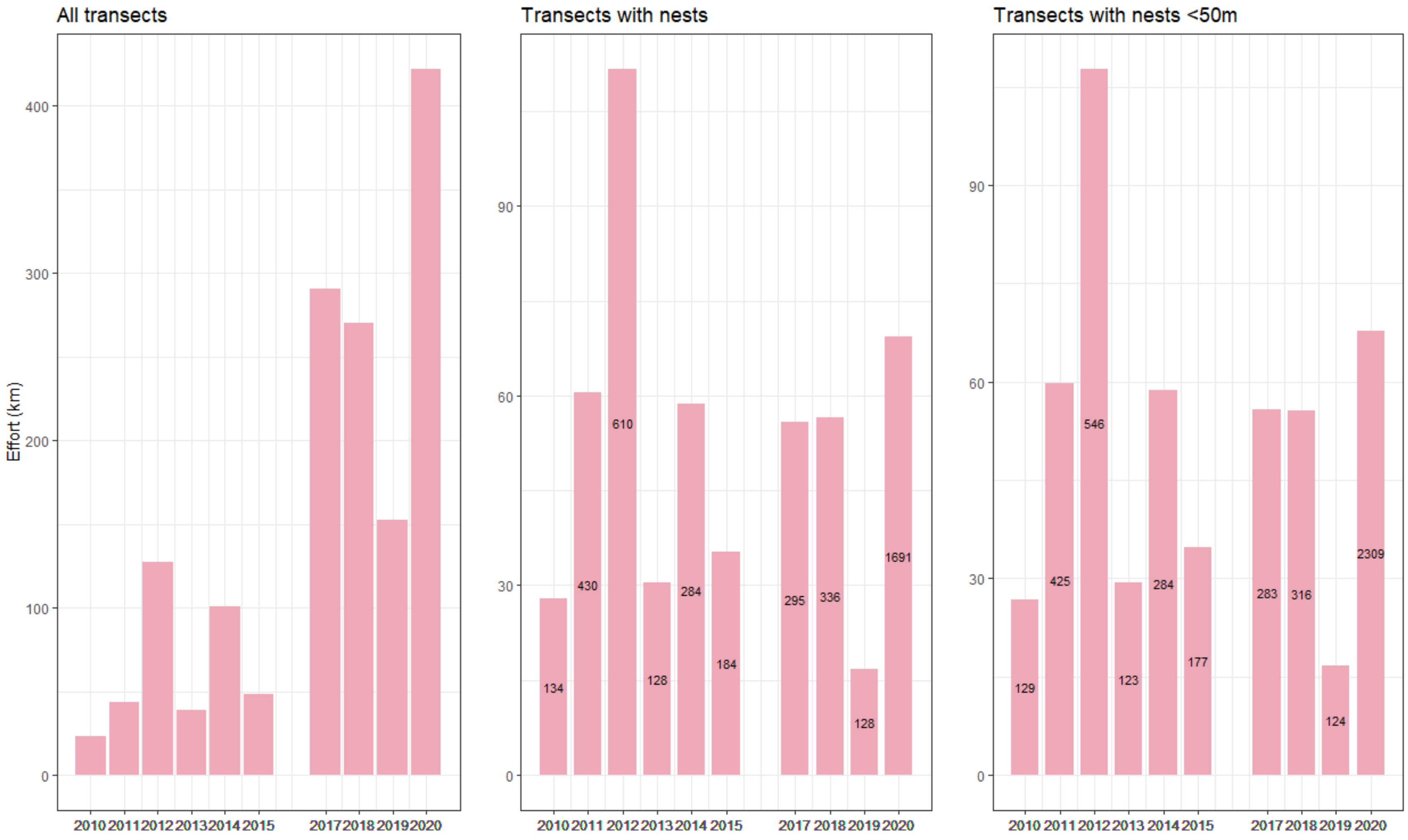

Figure S4. Sampling effort (km) between 2010-2020, except 2016, for 1) all transects, 2) only transects in which nests were observed, and 3) only transects in which nests were observed at a distance <50 m. The numbers in the bars correspond to the number of nests observed.

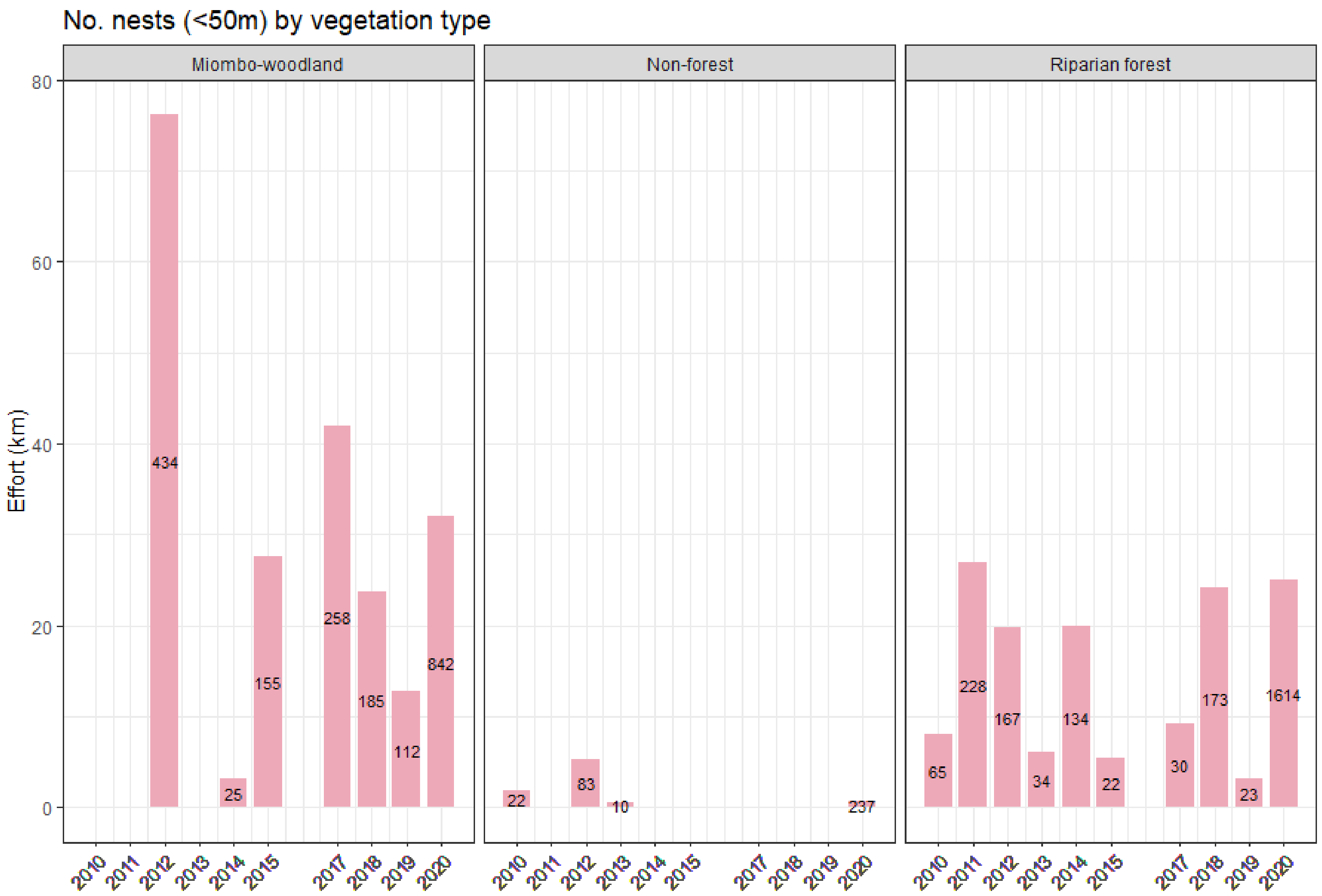

Figure S5. Sampling effort (km) and number of nests (truncated at a distance <50m; numbers inside bars) recorded in each vegetation type between 2010-2020, except 2016.

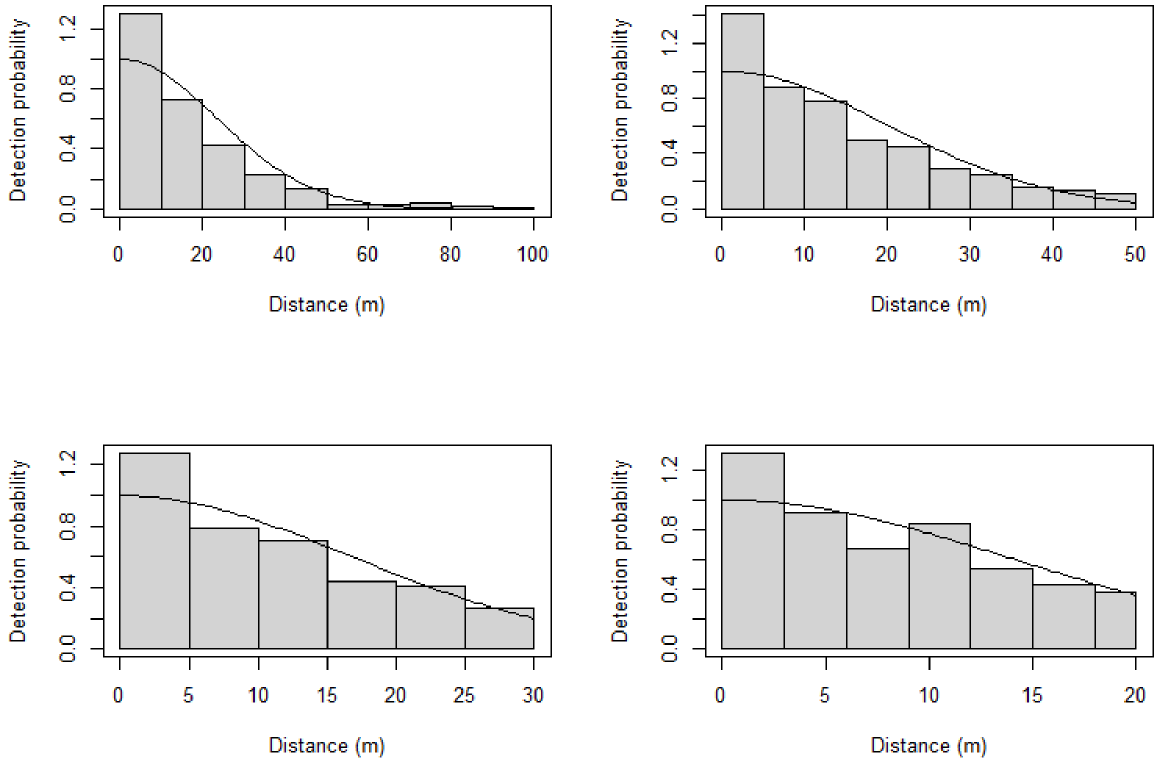

Figure S6. Dectection functions (half-normal key function) superimposed on histograms of nest distances truncated at 100 m, 50 m, 30 m and 20 m.

Table S4. Results of the half-normal key function without adjustments by using different truncation distances to nest data.

Truncation (m)	No. nests	Average detection probaility	Coeficient of Variation
100	4212	0.29	0.01
50	4057	0.50	0.01
30	3597	0.65	0.01
20	2924	0.74	0.02

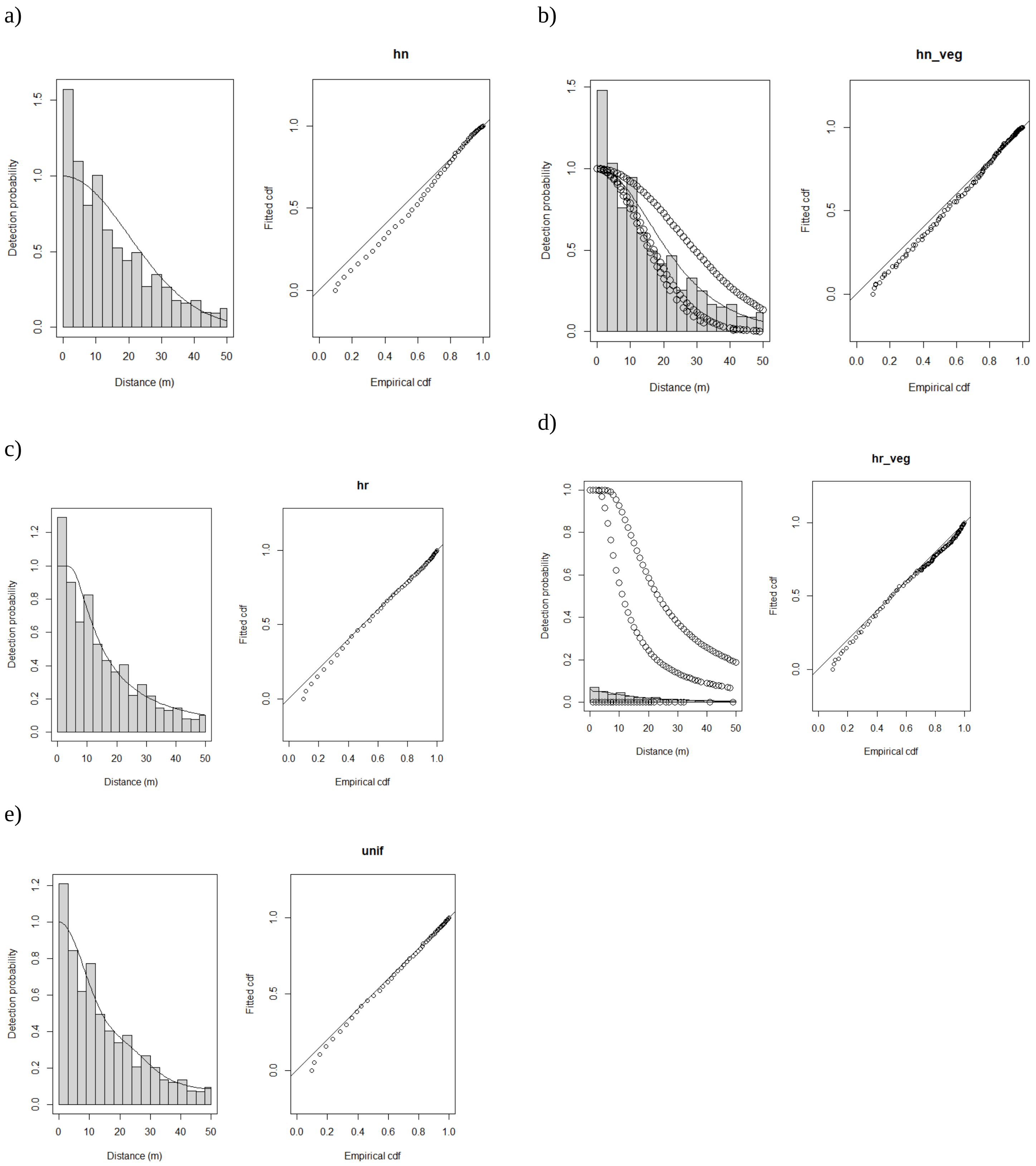

Figure S7. Candidate detection function models to select the best model: a) half-normal, b) half-normal with vegetation type, c) hazard-rate, d) hazard-rate with vegetation type, e) uniform. The first plot represents the estimated detection function superimposed onto a histogram of the observed distances truncated <50 m, and the second plot is a quantile-quantile (QQ) plot to assess how well a certain detection function fits the data using exact distances.

Table S5. Comparison of candidate detection functions of the observed distances truncated <50 m based on AIC and goodness of fit test (Cramer von Mises (C – vM) and respective p-value). The average detectability and respective coefficient of variation (CV) are also shown.

Key function	Formula	C - vM	p-value	Detectability (average)	Detectability (CV)	ΔAIC
Half-normal	~vegetation type	6.25	0.03e-02	0.47	0.011	0.0
Hazard-rate	~vegetation type	3.29	0.18e-07	0.02	0.091	617.3
Uniform with cosine adjustment term of order 1,2,3,4,5	NA	2.04	0.10e-06	0.38	0.025	842.6
Hazard-rate	~1	2.39	0.18e-07	0.41	0.025	874.9
Half-normal	~1	9.73	0.28e-02	0.50	0.011	1016.5

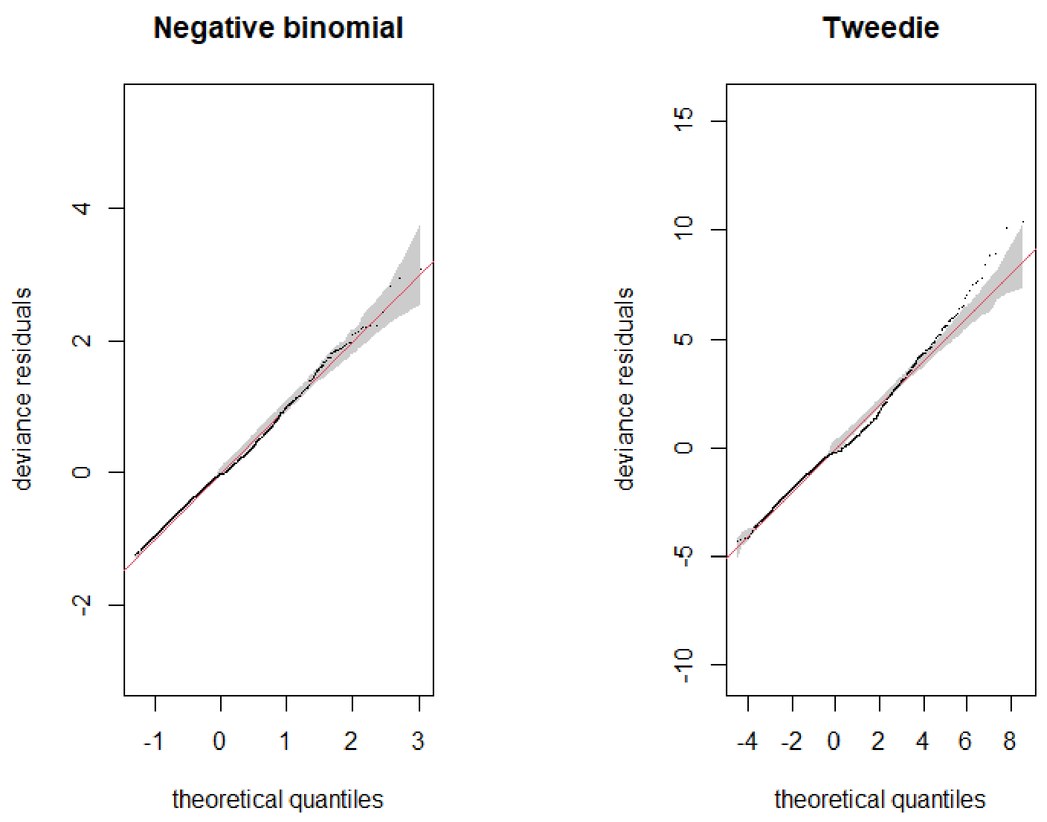

Figure S8. Residual quantile-quantile (QQ) plots of the model with negative binomial (left) and Tweedie (right) family distributions.

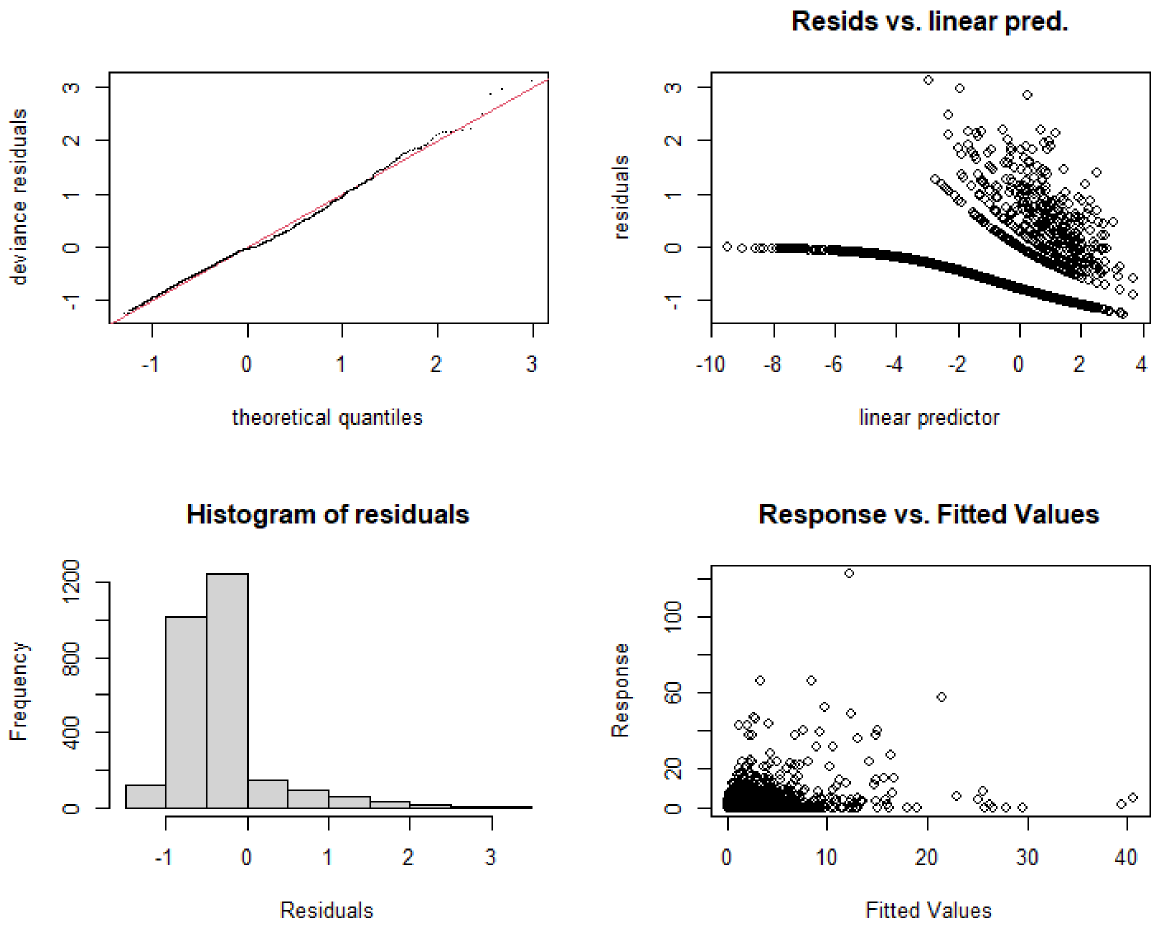

Figure S9. Diagnostic plots for the best model.

Table S6. Model results for both distribution families.

Response	AIC	Δ AIC	REML	Deviance explained (%)
Negative binomial (p=0.148)	4933.6	0	2503.3	43.4
Tweedie (p=1.422)	5044.9	111.3	2556.3	41.3

Table S7. Results for several negative binomial models.

Model	Terms	Overdispersion parameter	AIC	Δ AIC	REML	Deviance explained (%)
No veg type	s(year,k=7)+s(long,lat)+ti(long,year)+ti(lat,year)+ti(long,lat,year)+ s(annual temp)+s(distslope)+ s(elev)+log(hum)+s(distfor)	0.146	4938.7	7.8	2505.8	43.2
All variables	s(year,k=7)+s(long,lat)+ti(long,year)+ti(lat,year)+ti(long,lat,year)+ s(annual temp)+s(distslope)+ s(elev)+log(hum)+s(distfor)+ s(veg, k=2)	0.148	4933.6	2.7	2503.4	43.4
No year	s(long,lat)+ti(long,year)+ti(lat,year)+ti(long,lat,year)+ s(annual temp)+s(distslope)+ s(elev)+log(hum)+s(distfor)+ s(veg, k = 2)	0.148	4932.1	1.2	2502.9	43.6
No long or lat and year	s(year,k=7)+s(long,lat)+ti(long,lat,year)+s(annual temp)+s(distslope)+ s(elev)+log(hum)+s(distfor)+ s(veg, k = 2)	0.147	4931.8	0.9	2504.0	43.1
No temp. range	s(year,k=7)+s(long,lat)+ti(long,year)+ti(lat,year)+ti(long,lat,year)+ s(distslope)+ s(elev)+log(hum)+s(distfor)+ s(veg, k = 2)	0.148	4930.9	0	2502.9	43.5

s: smooth term, k= largest complexity for the smooth term, long: longitude, lat: latitude, annual temp: annual temperature, distslope: distance to slopes, elev: elevation, hum: human population density, distfor: distance to forests, veg: vegetation type.

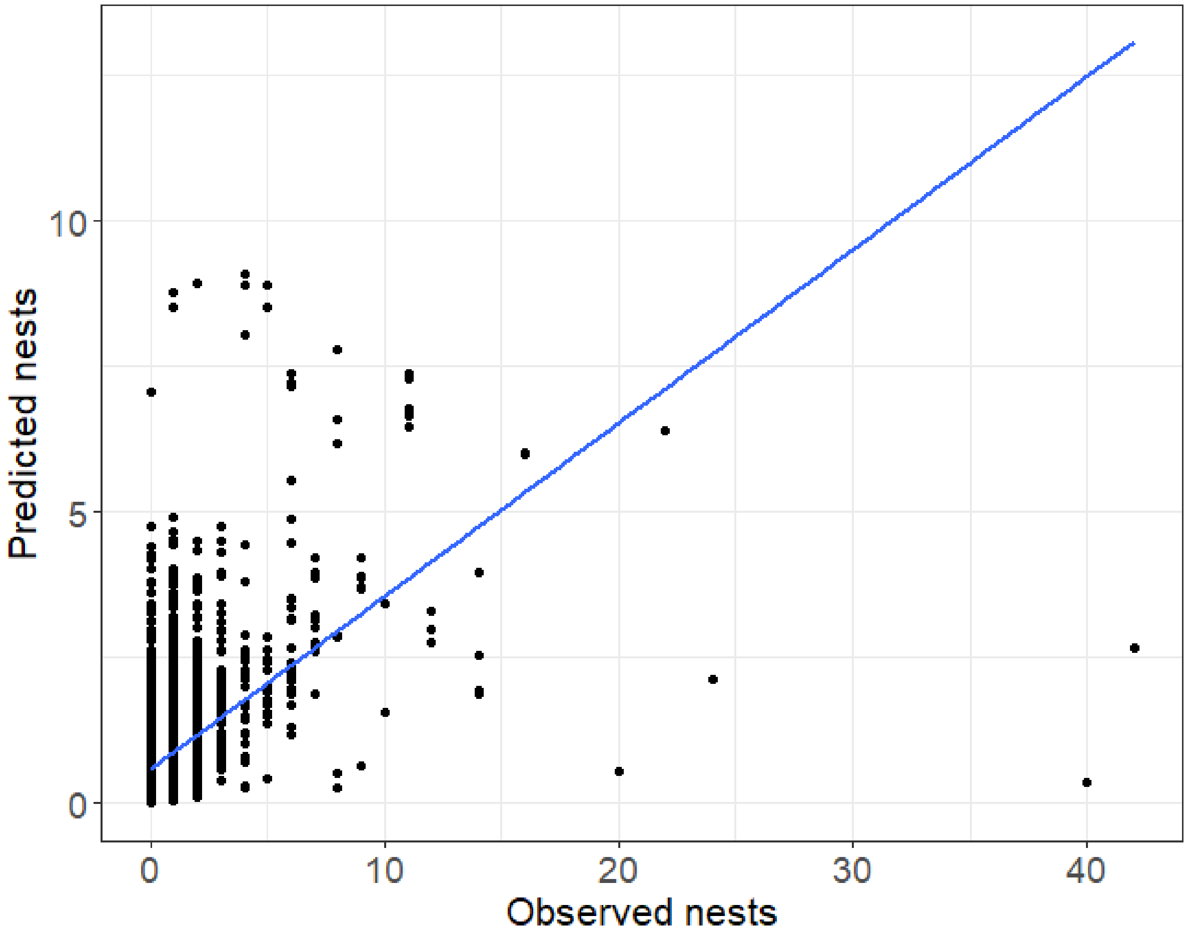

Figure S10. Comparison of the predicted nest abundance from 10-fold cross validation and the observed nest abundance. The blue dashed line represents the regression line.

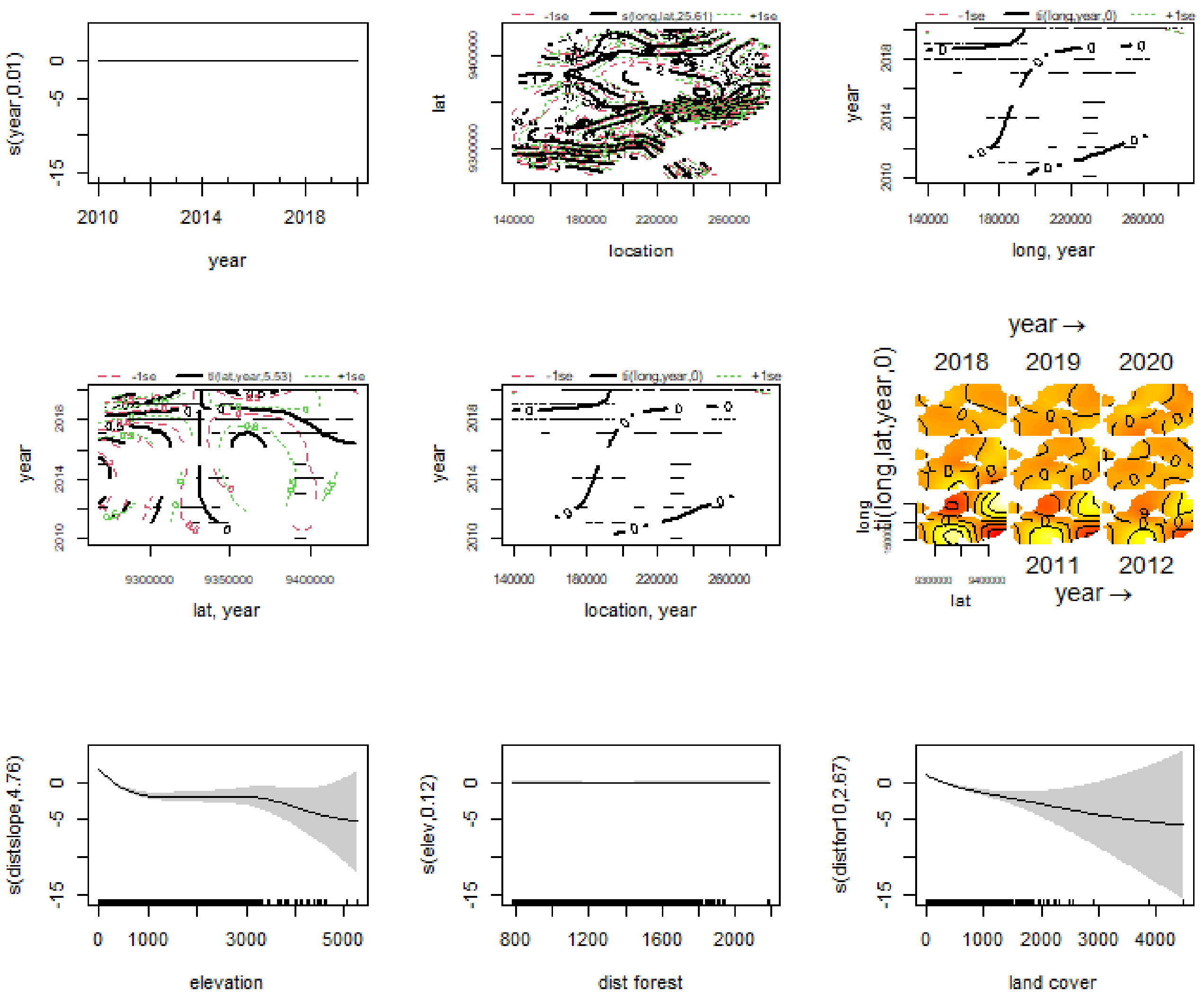

Figure S11. Plots of the smooth functions of the model predictors. Shaded bands indicate 95% confidence intervals. Numbers on the y- axis represent the effective degrees of freedom of the term (1 corresponds to a linear term). The rug ticks at the bottom represent the coverage of the values of each predictor in the study region.

References

Collins, D. A., & McGrew, W. C. (1988). Habitats of three groups of chimpanzees (Pan troglodytes) in western Tanzania compared. *Journal of Human Evolution*, *17*(6), 553–574. https://doi.org/10.1016/0047-2484(88)90084-X

Hansen, M. C., Potapov, P. V, Moore, R., Hancher, M., Turubanova, S. A., Tyukavina, A., … Townshend, J. R. G. (2013). High-resolution global maps of 21st-century forest cover change. *Science*, *342*(6160), 850–853. https://doi.org/10.1126/science.1244693

Stewart, F. A., Piel, A. K., & McGrew, W. C. (2011). Living archaeology: artefacts of specific nest site fidelity in wild chimpanzees. *Journal of Human Evolution*, *61*(4), 388–395. https://doi.org/10.1016/j.jhevol.2011.05.005

Zamma, K., & Makelele, M. (2012). Comparison of the longevity of chimpanzee beds between two areas in the Mahale Mountains National Park, Tanzania. *Pan African News News*, *19*(2).
